# Chest wall perforator flaps for breast reconstruction: international survey on attitudes and training needs

**DOI:** 10.1093/bjs/znad145

**Published:** 2023-06-01

**Authors:** Andreas Karakatsanis, Malin Sund, Nicola Rocco, Jill R Dietz, Ashutosh Kothari, Mustapha Hamdi, Yazan A Masannat, Peter A Barry

**Affiliations:** Department for Surgical Sciences, Uppsala University, Uppsala, Sweden; Section for Breast Surgery, Department of Surgery, Uppsala University Hospital, Uppsala, Sweden; Department of Surgical and Perioperative Sciences, Umeå University, Umeå, Sweden; Department of Surgery/CLINICUM, University of Helsinki and Helsinki University Hospital, Helsinki, Finland; G.Re.T.A. Group for Reconstructive and Therapeutic Advancements Fondazione ETS, Naples, Italy; Department of Advanced Biomedical Sciences, University of Naples ‘Federico II’, Naples, Italy; Breast Surgeon, Cleveland, Ohio, USA; Department of Breast Surgery, Guy’s and St Thomas’ NHS Foundation Trust, London, UK; Life Sciences and Medicine, Kings College, London, UK; Plastic and Reconstructive Surgery Department, Brussels University Hospital, Brussels, Belgium; Breast Unit, Aberdeen Royal Infirmary, NHS Grampian, Aberdeen, UK; iBreastBook, Aberdeen, Scotland; School of Medicine, University of Aberdeen, Aberdeen, UK; Department of Breast Surgery, Royal Marsden NHS Foundation Trust, Sutton, UK

## Abstract

**Background:**

Volume replacement using chest wall perforator flaps (CWPFs) is a promising technique to reduce mastectomy rates without sacrificing function or aesthetics. Owing to limited availability of the technique, only a minority of patients currently have access to CWPF procedures.

**Methods:**

An international web-based survey was disseminated through social media, dedicated webpages, and national and international societies for breast surgery. The survey explored surgeons’ attitudes towards CWPFs and their perceived training needs.

**Results:**

Of 619 respondents, 88.4 per cent agreed that CWPF surgery was desirable, with one-third offering it and performing a median of 10 (i.q.r. 5–15) procedures annually. They were more likely to be senior (OR 1.35, 95 per cent c.i. 1.18 to 1.55; *P* < 0.001), with formal oncoplastic training (OR 4.80, 3.09 to 7.48; *P* < 0.001), and working in larger units (OR 1.18, 1.03 to 1.35; *P* = 0.018) with a free-flap (OR 1.62, 1.06 to 2.48; *P* = 0.025) or CWPF (OR 3.02, 1.87 to 4.89; *P* < 0.001) service available. In cluster and latent class analysis, none showed high cohesion with performance of CWPF surgery.

**Conclusion:**

There is a discrepancy between perceived importance and availability of CWPF surgery, indicating that optimal training is needed.

## Teaser

Chest wall perforator flap reconstruction is an attractive oncoplastic technique that still needs to be standardized and disseminated. In an international survey of 620 respondents across the world, it was seen that, although most surgeons feel that the technique is important, very few are offering it and the results suggested that there is no clear profile of core skills that can lead to mastering it. The results of this survey highlight the knowledge gaps and the learning priorities, providing a basis for future standardization and consensus.

## Introduction

Breast-conserving therapy has equivalent survival to mastectomy in landmark randomized trials^1,2^. Observational data suggest that it might even confer a survival advantage^3^, with improved survivorship (satisfaction, function, and health-related quality of life) over mastectomy^4–6^. Oncoplastic breast-conserving therapy (OPBCT) facilitates removal of larger lesions and avoidance of mastectomy, while achieving excellent functional and aesthetic outcomes. The main reconstructive principles are volume displacement (reshaping by mammoplasty)^7^ or replacement, often with chest wall perforator flaps (CWPFs) (*Fig. S1*)^8,9^. The latter have not been widely adopted^10^. The lack of uniform perception of indications, techniques, and outcomes reflects a lack of standardized surgical training^1^, despite achievable oncological, functional, and aesthetic outcomes^12^. In the absence of uniformity and consensus^13^, understanding surgeon attitudes and needs in CWPF reconstruction education/training is a necessary first step. This study aimed to assess the perceived knowledge of CWPF surgery among surgeons of different background, specialty, and experience, practising in diverse settings; to define knowledge gaps and educational priorities; and to assess the optimal mode of education.

## Methods

The PERDITA (PERforator flaps: Doctors needs In Training and Attitudes) survey was conceived after a dedicated webinar (iBreastBook, 20/11/2021) on CWPFs. The survey was developed on Microsoft^®^ Forms (Office 365) (Microsoft, Redmond, WA, USA), and disseminated electronically through a link/QR code through social media (Facebook, Twitter, LinkedIn, WhatsApp), newsletters of groups (iBreastBook, Group for Reconstructive and Therapeutic Advancements (G.Re.T.A.), SENATURK), and national and international societies for breast/plastic surgery, surgical oncology, mastology, and gynaecology/senology, to ensure representation and diversity. Responses were stored on a safe server at Uppsala University Hospital. The survey was launched on 13 December 2021 and closed on 25 March 2022, to allow wide dissemination, and was advertised repeatedly to minimize non-response bias. The survey remains accessible online (https://forms.office.com/r/SXK2MffHCR) (*supplementary material*). Participants were asked to provide name and e-mail address, to avoid duplicates, exclude spam responses, and increase credibility. No reminders were needed as no personal invitations were sent. To avoid missing data, the items were marked as obligatory, except items addressed to those who already perform the technique or directed mainly at trainees.

The first part of the survey investigated baseline characteristics (demographics, surgical specialty, formal oncoplastic training, level of experience, respondents’ case mix, practice setting, unit annual caseload, and free-flap service availability). The second part examined whether respondents performed CWPF surgery, had attended relevant educational activities (online events, courses), and attitude/interest towards the technique. This was followed by a set of questions to elicit views on training, starting with whether respondents felt that every unit needs a CWPF service, whether every surgeon should be able to perform the procedure independently, and what the available training opportunities are. Then the surgeon’s status of knowledge and perception of the current literature and other resources were assessed (on a Likert scale from 1 to 10) regarding anatomy, technique, indications, and outcomes. The CWPF procedure was broken down into nine steps: candidate identification, flap design/markings, basic/vascular ultrasound imaging, preoperative perforator identification on radiology, intraoperative perforator identification and dissection, flap mobilization/placement, and wound closure. The participants were asked whether they felt they needed training for each of these steps (yes, no, or unsure). Eight different alternatives of learning resource were given (reading material, videos, webinars, courses, workshops, assisting in theatre, theatre supervision, and performing CWPF unsupervised), without limiting the number of responses. Afterwards, respondents were asked to rank these alternatives. The last few questions were about local training (whether it covers CWPF surgery, and the optimum number of procedures needed to show competency).

### Statistical analysis

An open, cross-sectional, random population sample survey was designed. Allowing for approximately 50 000 breast surgeons around the world and for a 50 per cent distribution on outcome of interest, a minimum of 382 responses was needed for a loss of estimate no larger than 5 per cent (Epi Info™ version 7.2.5.0). In sensitivity analyses of the sample size calculation, the number required for populations over 1 000 000 did not exceed 384.

Results were stratified by region, income by country, surgical discipline, level of expertise, and practice setting. Nominal variables were summarized as absolute numbers with percentages and 95 per cent confidence intervals, and ordinal variables as either numbers with percentages and 95 per cent confidence intervals, or median (i.q.r.). Associations were investigated using the χ^2^ test for unpaired data, and McNemar’s test for paired data. Medians were compared as appropriate (Mann–Whitney *U* test for two independent samples, Wilcoxon signed-rank test for related samples, Friedman test of medians for k samples, and Friedman’s 2-way ANOVA by ranks). Multivariable analyses by logistic regression with linearized standard errors and ordinal regression were undertaken if univariable analyses denoted statistical significance (*P* < 0.050). These were preceded by control for collinearity and overfitting. The outcomes are reported as β coefficients (logarithms) or exponentiated effect sizes (ORs with 95 per cent c.i.). Two-step cluster analyses and latent class analyses were undertaken to identify cohesion and hidden associations. The analyses were performed with SPSS^®^ version 28 (IBM, Armonk, NY, USA) and Stata^®^ version 17 (StataCorp, College Station, TX, USA). The manuscript was reported according to CROSS guidelines^14^.

## Results

### Baseline characteristics

A total of 638 responses were received. After removal of 3 spam messages and 16 duplicates, 619 responses were analysed. The respondents’ demographics are summarized in *Table S1*. The probability that the respondents’ unit offered CWPF surgery was associated with region, country income, healthcare setting, unit annual caseload, availability of free-flap plastic surgery service in the unit, surgical specialty, and formal oncoplastic training of the respondents. In multivariable logistic regression analysis, country income, healthcare setting, unit annual caseload, free-flap plastic surgery service in the unit, surgical specialty, and formal oncoplastic training retained significance (*Table 1*). No collinearity was present. Although free-flap plastic surgery service in the unit was the strongest predictor (OR 3.727), there was significant discordance regarding whether units offering free flaps also offer CWPF surgery (difference 24.5 (95 per cent c.i. 20.1 to 29.0) per cent; *P* < 0.001).

**Table 1:** Multivariable regression analysis of factors interacting with unit offering chest wall perforator flaps service

	OR	*P*
Region	1.01 (0.92, 1.11)	0.857
Country income*	0.61 (0.45, 0.83)	0.002
Surgical specialty†	0.81 (0.66, 0.98)	0.027
Oncoplastic training‡	1.67 (1.07, 2.61)	0.025
Experience	0.91 (0.78, 1.06)	0.221
Practice setting§	0.66 (0.49, 0.87)	0.004
Annual unit caseload¶	1.31 (1.13, 1.52)	<0.001
Free-flap plastic surgery service#	3.73 (2.37, 5.86)	<0.001

Values in parentheses are 95% confidence intervals.*Higher income relates to positive outcome. †Dedicated breast surgeon relates to positive outcome. ‡Oncoplastic training: ‘yes’ relates to positive outcome. §University/teaching hospital relates to positive outcome. ¶Higher annual caseload relates to positive outcome. #Free-flap plastic surgery service: ‘yes’ relates to positive outcome.

Regarding respondents’ perceived competence, eight options with different surgical techniques were provided as alternatives. With a median of 3 (i.q.r. 2–6) alternatives for the entire survey, 209 (33.8 per cent) performed CWPF surgery, ranking as fifth after wide local excision and mastectomy (453, 73.2 per cent), level I OPBCT (442, 71.4 per cent), therapeutic mammaplasty (411, 66.4 per cent), and implant-based reconstruction (333, 53.8 per cent). CWPF surgery tied with pedicled flaps for mastectomy reconstruction (209, 33.8 per cent) and was followed by free flaps for mastectomy reconstruction (38, 6 per cent) and ‘I do not perform any cases independently’ (29, 5 per cent).

Univariable analyses showed associations with region, surgical specialty, level of experience, practice setting, annual unit caseload, unit with free-flap service, unit with CWPF service, and formal oncoplastic training. In logistic regression analysis, those who responded that they perform CWPF reconstruction were more likely to be more experienced (attending/consultants), and to work in larger units with available free-flap service, CWPF service, and that had formal oncoplastic training (*Table 2*). Cluster analysis identified two clusters with a silhouette measure of cohesion and separation of 0.2 (poor to fair) (*Table S2*). The in-cluster predictor importance was 0.99, second to whether a unit offered CWPF surgery. Latent class analysis did not identify any associations between CWPF surgery and the other factors, nor within the latent classes. Respondents who perform CWPF surgery had been performing the technique for a median of 3 (i.q.r. 1–5) years and the median number of procedures per year was 10 (5–15); there were no factors interacting with these outcomes.

**Table 2:** Multivariable regression analysis of factors interacting with surgeon performing chest wall perforator flaps

	Univariable analysis				
	Frequencies		*P*	Logistic regression analysis	
	Yes	No		OR*	*P*
**Region**			0.003	1.05 (0.97, 1.14)	0.240
Europe	103 (37.7)	170 (62.3)			
Central Asia	9 (20.5)	35 (79.5)			
Middle East and North Africa	17 (20.2)	67 (79.8)			
Sub-Saharan Africa	2 (14.3)	12 (85.7)			
South Asia	22 (37.9)	36 (62.1)			
East Asia and Pacific	19 (41.3)	27 (58.7)			
North America	1 (7.7)	12 (92.3)			
Latin America and Caribbean	29 (43.3)	38 (56.7)			
Not provided/missing	1 (20.0)	4 (80.0)			
**Country income**			0.142		
High	115 (37.8)	189 (62.2)			
Upper middle	41 (26.1)	116 (73.9)			
Lower middle	48 (34.5)	91 (65.5)			
Low	2 (28.6)	5 (71.4)			
Not provided	3 (25.0)	9 (75.0)			
**Surgical specialty**			<0.001	0.90 (0.76, 1.06)	0.213
Dedicated breast surgeon	128 (47.4)	142 (52.6)			
General surgeon doing some breast surgery	21 (13.8)	131 (86.2)			
Plastic surgeon	29 (33.7)	57 (66.3)			
Surgical oncologist	26 (30.6)	59 (69.4)			
Gynaecologist	5 (19.2)	21 (80.8)			
**Level of experience**			<0.001	1.35 (1.18, 1.55)	<0.001
Consultant/attending > 10 years	101 (37.7)	167 (62.3)			
Consultant/attending 5–10 years	51 (41.5)	72 (58.5)			
Consultant/attending <5 years	39 (40.6)	57 (59.4)			
Fellow after completion of specialist training	14 (23.0)	47 (77.0)			
Registrar/senior trainee	4 (8.5)	43 (91.5)			
Junior trainee	0 (0)	24 (100)			
**Practice setting**			0.006	1.28 (0.99, 1.64)	0.057
University/teaching hospital	114 (36.0)	203 (64.0)			
Public healthcare hospital	40.0 (23.8)	128 (76.2)			
Private hospital	51 (40.5)	75 (59.5)			
≥ 1 or all of the above	4 (57.1)	3 (42.9)			
**Annual unit caseload**			<0.001	1.18 (1.03, 1.35)	0.018
<50	17 (18.3)	76 (81.7)			
50–100	25 (25.3)	74 (74.7)			
101–150	23 (29.1)	56 (70.9)			
151–300	56 (38.4)	90 (61.6)			
301–500	43 (45.3)	52 (54.7)			
> 501	42 (48.3)	45 (51.7)			
Don't know	3 (15.0)	17 (85.0)			
**Unit with free-flap reconstruction service**			<0.001	1.62 (1.06, 2.48)	0.025
Yes	138 (43.8)	177 (56.2)			
No	71 (23.4)	233 (76.6)			
**Unit with chest wall perforator service**			<0.001	3.02 (1.87, 4.89)	<0.001
Yes	95 (58.3)	68 (41.7)			
No	114 (25.0)	342 (75.0)			
**Formal oncoplastic training**			<0.001	4.80 (3.09, 7.48)	<0.001
Yes	170 (49.3)	175 (50.7)			
No	39 (14.2)	235 (85.8)			

Values are *n* (%) unless otherwise indicated; *values in parentheses are 95% confidence intervals.

### Current perceptions on CWPF training needs and outcomes

The majority of respondents (88.4 per cent) had a positive attitude towards the need for CWPF surgery. Most had attended a webinar rather than a course/workshop (77.5 *versus* 25.8 per cent; difference 51.7 (95 per cent c.i. 47.4 to 56.0) per cent; *P* < 0.001). Of those who had attended a webinar (483, 77.5 per cent), 185 (30.3 per cent) were intending to start CWPF surgery and the event motivated them even more, 146 (23.9 per cent) were already performing CWPF surgery and were interested to learn more, and 101 (16.5 per cent) were not intending to start, but the event motivated them. On the contrary, 39 (6 per cent) were intending to start but were dissuaded, whereas 12 (2 per cent) were not interested and the webinar did not change this. Of the 128 respondents (25.5 per cent) who had not attended any webinars, 108 (17.7 per cent) were interested in CWPF surgery, whereas the remaining 20 (3 per cent) were not. Overall, a webinar was more likely to motivate (20.9 per cent) than dissuade (8.1 per cent) surgeons to consider starting CWPF surgery (difference 12.8 (8.0 to 17.7) per cent; *P* < 0.001).

There was significant discordance in the responses on whether all breast units or all breast surgeons should offer CWPF reconstruction (*Table S3*). The difference persisted even when the outcomes were dichotomized (yes *versus* all others), with 67 per cent for all units *versus* 52.5 per cent for all surgeons (difference 14.5 (10.8 to 18.4) per cent; *P* < 0.001).

Participants further responded on their own perception of patient and surgeon satisfaction with CWPF surgery, scoring a median of 8 (i.q.r. 7–9) of 10 for both questions. Interestingly, there was significant discordance on these paired items (*P* < 0.001, Wilcoxon signed-rank test), with 52.9 per cent ties, 35.6 per cent higher patient satisfaction, and 11.5 per cent higher surgeon satisfaction, an outcome not associated with any of the input variables.

Respondents provided similar Likert ratings on their own perceived knowledge level of CWPF anatomy, technique, indications/contraindications, and outcomes, as well as the adequacy of literature and learning resources on these subjects (*Table 3*). Again, the median values at group level for each question were similar, but there was significant discordance among respondents. The majority felt that their knowledge level in all four items was lower than that of the available resources, depending primarily on whether the respondents performed CWPF surgery (*Table S4*). This retained significance in ordinal multivariable regression analysis.

**Table 3:** Surgeons’ perception of personal knowledge of chest wall perforator flap anatomy, technique, indications, and outcomes related to perceived adequacy of learning sources

	Perceived adequacy of literature/resources*	Self-perceived knowledge*	Ties	Positive difference (literature better)	Negative difference (surgeon better)	*P‡*	Significant factors in ordinal regression	Coefficient†	*P*⁑
Anatomy	7 (5–9)	7 (5–8)	210 (33.9)	250 (40.4)	159 (25.7)	<0.001	Surgeon not performing CWPF surgery	0.54 (0.19, 0.88)	0.002
Technique	7 (5–8)	6 (4–8)	176 (28.4)	296 (47.8)	147 (23.8)	<0.001	Dedicated breast surgeon	−0.75 (−1.49, −0.02)	0.045
							Surgeon not performing CWPF surgery	0.84 (0.49, 1.19)	<0.001
Indications	7 (5–8)	7 (5–8)	222 (35.9)	222 (35.9)	175 (28.2)	<0.001	Surgeon not performing CWPF surgery	1.05 (0.70, 1.40)	<0.001
Outcomes	7 (5–8)	7 (5–8)	239 (38.6)	207 (33.3)	173 (27.9)	0.022	Consultant/attending <5 years	−0.63 (−1.14, −0.18)	0.016
							Surgeon not performing CWPF surgery	1.04 (0.68, 1.39)	<0.001

Values are *n* (%) unless otherwise indicated; *values are median (i.q.r.) score on Likert scale from 1 to 10; †values in parentheses are 95% confidence intervals. Ordinal regression (logit) was used to identify the factors associated with a difference. CWPF, chest wall perforator flap. ‡: Wilcoxon paired signed rank test; ⁑: ordinal regression (logit)

Finally, in answer to the question about whether their formal training included CWPF surgery, the response was ‘no’ from 414 participants (66.9 per cent), followed by ‘yes’ (106, 17.1 per cent), ‘unsure’ (39, 6.3 per cent), and missing (60, 9.7 per cent). The only factor associated with exposure to CPWF surgery was formal oncoplastic training (78 *versus* 28 (24.7 *versus* 11.5 per cent); *P* < 0.001). The majority (226, 54.6 per cent) also responded that training will not/did not ensure acquisition of the necessary experience to perform CWPF surgery. Those who responded that their training was/will be sufficient were more likely to be plastic surgeons (49.3 per cent), gynaecologists (47.8 per cent), surgical oncologists (46.5 per cent), dedicated breast surgeons (36.7 per cent), and general surgeons (25.0 per cent) (*P* = 0.020, χ^2^ test), and those with formal oncoplastic training regardless of core discipline (45.5 *versus* 26.3 per cent; *P* < 0.001, χ^2^ test). In logistic regression, only formal oncoplastic training retained significance (OR 3.08, 95 per cent c.i. 1.78 to 5.32; *P* < 0.001).

### Perceptions on training needs

With regard to responses to the nine different items related to the CWPF procedure (candidate identification, flap design/markings, basic/vascular ultrasound, preoperative perforator identification on radiology, intraoperative perforator identification, flap mobilization/placement, and wound closure), a minimum of 60 per cent responded that they need training in all, except wound closure, whereas the frequency of response denoting uncertainty was low (below 9 per cent) for all items (*Table 4*). Responses differed depending on whether the respondents were performing CWPF surgery or not (*Table S5*). Even among respondents who were undertaking CWPF surgery, a significant proportion stated that they needed training in some or more of the steps, the response rate ranging from 32.8 per cent for wound closure to 60.8 per cent for preoperative perforator identification.

**Table 4:** Identification of needs in training in different technical aspects of chest wall perforator flap reconstruction

	Yes	No	Unsure
Do you need training in identifying CWPF candidates?	399 (64.9)	165 (26.7)	55 (8.9)
Do you need training in flap design/markings?	477 (77.1)	121 (19.5)	21 (3.4)
Do you need training in basic ultrasound?	367 (59.3)	222 (35.9)	30 (4.8)
Do you need training in vascular (Doppler) ultrasound?	443 (71.6)	151 (24.4)	25 (4.0)
Do you need training in radiology review for perforator identification?	470 (75.9)	105 (17.0)	44 (7.1)
Do you need training in raising a flap?	421 (68.0)	157 (25.4)	41 (6.6)
Do you need training in perforator dissection?	481 (77.7)	110 (17.8)	28 (4.5)
Do you need training to place the flap in the cavity?	413 (66.7)	169 (27.3)	37 (6.0)
Do you need training in wound closure?	278 (44.9)	308 (49.8)	33 (5.3)

Values are *n* (%). CWPF, chest wall perforator flap.

The participants’ responses regarding the eight different alternative learning resources are summarized in *Table 5*. The median number of alternatives chosen was 5 (i.q.r. 3–7, range 1–8) for the entire survey. The results suggest that, although there was a clear view of what is most important (read about anatomy, technique, etc.), there was no alternative ranking as the best solution. The alternatives with intermediate solutions (webinar, course, workshop) were without a clear ranking, but there was consensus on the least effective alternative, that is to perform procedures unsupervised.

**Table 5:** Participants’ responses regarding training modalities by frequency and ranking

	Frequencies		Rank			
	Yes	No	Crude rank	%	Median	Mean rank*
Read about anatomy, technique, etc.	449 (72.5)	170 (27.5)	1	48.1	2	2.80
Watch videos	462 (74.6)	157 (25.6)	2	29.7	3	3.69
Attend a web-based event	382 (61.7)	237 (38.3)	3	24.7	4	4.36
Attend a course	410 (66.2)	209 (33.8)	4	31.2	4	3.85
Attend a workshop	433 (70.0)	186 (30.0)	5	26.5	4	3.99
Attend/assist in the theatre	415 (67.0)	204 (33.0)	6	34.6	5	4.43
Perform cases supervised	423 (68.3)	196 (31.7)	7	46.2	7	5.31
Perform cases unsupervised	68 (11.0)	551 (89.0)	8	85.8	8	7.56

Friedman’s two-way ANOVA by ranks.

## Discussion

An increasing number of breast units are offering CWPF surgery to complement other oncoplastic techniques and further reduce mastectomy rates^15,16^. This is particularly important in health service delivery in systems where reconstructive options are limited by either human resources or materials, or both^15,17^. Breast surgeons globally have several training pathways, most commonly through core training in general surgery, surgical oncology, gynaecology/senology, and plastic/reconstructive surgery. The technical skills required for CWPF surgery have, however, traditionally been in the domain of plastic and reconstructive surgical training. Interestingly, the PERDITA survey indicated a global positive attitude from all major world regions, independent of baseline characteristics. Yet, it seems that only a minority of surgeons offer the service.

The strongest predictor of a CWPF service within a unit was the presence of a free-flap whole-breast reconstruction service, but there was still significant discordance between the availability of both. A probable explanation is that surgeons unfamiliar with the technique not only do not offer it, but often cannot identify appropriate candidates, as the survey results demonstrated. In terms of perceived procedural competency within the breast oncoplastic repertoire, CWPF surgery ranked fifth, surpassed only by free-flap reconstruction in terms of perceived complexity. This illustrates that, regardless of background characteristics or positive attitude regarding the need for CWPF surgery and its clinical value, it is a challenging technique.

The results were conflicting regarding the most probable profile of those undertaking CWPF procedures. Those who already performed CWPF surgery were more senior (attending/consultant level), with formal oncoplastic training, and working in larger units with a free-flap service. Cluster and latent class analyses, however, showed poor separation between those who did and those did not practice CWPF surgery in terms of these predictors. This finding suggests that experience, exposure, and a particular skillset are probably necessary, but not sufficient conditions. Among those performing CWPF surgery, the median caseload was only 10 per year with median experience of only 3 years. This probably accounts for attitudes towards all *versus* selected surgeons in a unit being trained to perform such procedures. Clearly, there is concern that the greater the number of surgeons undertaking CWPF surgery, the more individual experience is diluted. On the other hand, there was significant discordance between desire for all surgeons in a given unit to undertake CWPF procedures *versus* attitudes towards all units offering them. This finding suggests that, apart from personal interest, many units would probably aim to allocate CWPF surgery to specialized surgeons.

While the evolution of practice from initial descriptions^9^ has been focused in a few large centres, a need for CWPF dissemination has reached critical level. Yet, there is no formalized training methodology and equally no formalized assessment of qualifications, competencies, or outcomes. The establishment and standardization of an independent curriculum on oncoplastic surgery is one of the main priorities, with important initiatives from national and international societies and working groups^18,19^. In the UK, this is evidenced by its incorporation into the 2022 Joint Committee for Surgical Training oncoplastic breast curriculum^20^. The need for formalized training is exemplified in this survey, as two-thirds of respondents with formal oncoplastic training had not been exposed to CWPF surgery, despite oncoplastic training being the only factor associated with exposure to the procedure. Additionally, 36.5 per cent of respondents felt that training did not or would not necessarily translate into adequate experience in performing CWPF surgery.

Standardizing an ideal curriculum is challenging. Apart from international, national, and local variances, meeting individualized needs has to account for any existing baseline discordance between one’s perceived knowledge and the perceived adequacy of literature and existing resources. Specifically, in CWPF surgery, landmark literature on anatomy, technique, indications, and outcomes is yet to be agreed upon, whereas perception of self-knowledge depends mainly on whether one is undertaking the procedure. Despite respondents rating both landmark literature and perception of self-knowledge for each domain (anatomy, technique, indications, and outcomes) comparably at a cohort level, that was not the case at an individual level. When respondents were asked to rank a deconstruction of the skillset needed to perform CWPF surgery, over 60 per cent stated the need for training in each of the skill components apart from wound closure. The more technical and skill-specific components (flap design/marking and perforator dissection) attracted the highest percentages. Notably, this also included large numbers of those who already perform the procedure. Respondents were open to all methods of training delivery. There was consensus on the prerequisite of a solid theoretical background, and, conversely, they reassuringly ranked unsupervised performance of the procedure as least desirable. There was, however, no clear ranking preference for any other single method including virtual and hands-on courses/workshops, an observation in line with subspecialization training in other surgical disciplines^21,22^.

Survey studies have certain limitations. In the PERDITA survey, the response items were marked as obligatory, to avoid missing data. Additionally, defining how any non-response bias might have skewed the results is extremely challenging for an open survey. The number of responses, however, significantly exceeded the minimum required sample size. Finally, even though responses from the USA were not proportionate to the number of breast surgeons, probably reflecting lack of dissemination through formal channels, the otherwise wide geographical distribution ensures that the results reflect a significant majority of surgeons working in breast surgery, regardless of core specialty, experience, or professional setting.

## Supplementary Material

znad145_Supplementary_Data

## Data Availability

The full data set of the survey will be available upon reasonable request to A.K. following completion of the PERDITA project and after a data-sharing agreement has been signed.
