# Supplementary material for: Chest wall perforator flaps for breast reconstruction: international survey on attitudes and training needs
Source: Br J Surg. 2023 Jun 1;110(8):966–72. doi: 10.1093/bjs/znad145 (PMC10361678; doi:10.1093/bjs/znad145)
Supplement: znad145_Supplementary_Data [file znad145_supplementary_data.docx]

**An international survey on attitudes and training needs in chest wall perforator flaps for breast reconstruction.**

Andreas Karakatsanis^1, 2^, Malin Sund^3, 4^, Nicola Rocco^5, 6^, Jill R. Dietz^7^, Ashutosh Kothari^8,9^, Mustapha Hamdi^10^, Yazan A. Masannat^11,12,13^ *, Peter A. Barry^14^ *

1. Department for Surgical Sciences, Uppsala University, Uppsala, Sweden.

2. Section for Breast Surgery, Department of Surgery, Uppsala University Hospital, Uppsala, Sweden.

3. Department of Surgical and Perioperative Sciences, Umeå University, Sweden.

4. Department of Surgery/CLINICUM, University of Helsinki and Helsinki University Hospital, Finland.

5. G.Re.T.A.; Group for Reconstructive and Therapeutic Advancements Fondazione ETS, Italy.

6. Department of Advanced Biomedical Sciences, University of Naples "Federico II", Naples, Italy.

7. Breast Surgeon, Cleveland, Ohio, United States of America.

8. Department of Breast Surgery, Guy’s & St. Thomas’ NHS Foundation Trust, London.

9. Life Sciences and Medicine, Kings College, London, United Kingdom.

10. Plastic and Reconstructive Surgery Department, Brussels University Hospital, Brussels, Belgium.

11. The Breast Unit, Aberdeen Royal Infirmary, NHS Grampian, Aberdeen, Scotland, United Kingdom

12. iBreastBook, United Kingdom (www.ibreastbook.com)

13. School of Medicine, University of Aberdeen, Aberdeen, Scotland, United Kingdom

14. Department of Breast Surgery, Royal Marsden NHS Foundation Trust, Sutton, United Kingdom

**Corresponding author:** Andreas Karakatsanis

**Address:** Department for Surgical Sciences, Uppsala University, Sjukhusvägen ing 70, SE 751 85, Uppsala Sweden.

**ORCID:** 0000-0003-3622-3575

**Email:** andreas.karakatsanis@surgsci.uu.se

**Twitter:** @KarakatsanisA

**Author Notes:** Yazan Masannat and Peter Barry contributed equally to the manuscript

Preliminary results of this project have been presented at the Association of Breast Surgeons Conference 2022 (European journal of surgical oncology: the journal of the European Society of Surgical Oncology and the British Association of Surgical Oncology 48(5):e191 DOI: 10.1016/j.ejso.2022.03.028)

**Funding:** No funding available for the present project

**Conflict of Interest Statement:** All authors declare no conflict of interest related to the present project

**Data sharing statement:** The full dataset of the survey will be available upon reasonable request after the completion of the PERDITA project and after a data sharing agreement, after contact with the corresponding author (Andreas Karakatsanis, e-mail address: andreas.karakatsanis@surgsci.uu.se)

**Supplementary Materials - Index**

| **Supplementary Methods** |  |
| --- | --- |
| The PERDITA survey | *pag. 3* |
| **Supplementary Figures and Tables** |  |
| Figure S1: Figure of CWPF reconstruction | *pag. 18* |
| Table S1: Characteristics of survey population | *pag. 19* |
| Table S2: Cluster analysis for the profiling of surgeons performing CWPF independently | *pag. 20* |
| Table S3: Crosstabulation between the need for every Unit vs every Surgeon to perform CWPF. | *pag. 21* |
| Table S4: Crosstabulation of scoring differences between respondents’ own perceived level of knowledge and the available literature/learning sources. | *pag. 22* |
| Table S5: Crosstabulation of training needs in different technical steps and whether the respondents independently perform CWPF. | *pag. 23* |
|  |  |
|  |  |
|  |  |
|  |  |
|  |  |
|  |  |

**Supplementary Methods**

PERforator flaps: Doctor needs In Training and Attitudes. The PERDITA Survey

Dear colleagues,

Chest wall perforator flaps are a part of oncoplastic and reconstructive volume replacement techniques that have gained interest and popularity in recent years. As such, there is an urgent need to understand how surgeons think about perforator flaps. How popular is the technique? What is the interest for it? What are the knowledge gaps and the learning opportunities and how do we all, surgeons, feel that our training needs will meet optimal patient outcomes?

With this specifically engineered survey, we aim to systematize and summarize current status, define training needs and research priorities and discuss structured approaches for the development of a toolbox for perforator flap reconstuction.

The survey is designed to take 8-9 minutes of your time. Please respond as you feel appropriate; there is no right or wrong! Your response is important, as it will help us understand training priorities, define knowledge gaps and report on need for focused research. The results will be announced as soon as the dataset is analyzed and a written

report is expected in a peer-reviewed journal. Participation is voluntary and anonymous. Entering your email address will allow us to avoid duplicates, thereby enhancing the quality

of the results.

With appreciation,

Yazan Masannat, Andreas Karakatsanis and Peter Barry
Background

* Required

The following items will help us understand your professional background

1.

Please enter your email-address. Its sole purpose is to allow us to avoid duplicates *

[https://forms.office.com/pages/designpagev2.aspx?lang=en-US&origin=OfficeDotCom&route=Start&subpage=design&id=bCpreHvSf02cWcwEq](https://forms.office.com/pages/designpagev2.aspx?lang=en-US&amp;origin=OfficeDotCom&amp;route=Start&amp;subpage=design&amp;id=bCpreHvSf02cWcwEq)… 1/15

2022-12-18 10:16 PERforator flaps: Doctor needs In Training and Attitudes. The PERDITA Survey

2. In which country are you currently practising? *

3. What is your specialty? *

General Surgeon doing some breast surgery

Dedicated Breast Surgeon without any other commitments

Plastic Surgeon

Gynaecologist

Surgical Oncologist

4. What is your specialty? *

General Surgeon doing some breast surgery

Dedicated Breast Surgeon without any other commitments

Plastic Surgeon

Gynaecologist

Surgical Oncologist

5.

Have you had formal oncoplastic training (including, but not limited, to a formal fellowship?) *

Yes

No

[https://forms.office.com/pages/designpagev2.aspx?lang=en-US&origin=OfficeDotCom&route=Start&subpage=design&id=bCpreHvSf02cWcwEq](https://forms.office.com/pages/designpagev2.aspx?lang=en-US&amp;origin=OfficeDotCom&amp;route=Start&amp;subpage=design&amp;id=bCpreHvSf02cWcwEq)… 2/15

2022-12-18 10:16 PERforator flaps: Doctor needs In Training and Attitudes. The PERDITA Survey

6. What is your current level of expertise? *

Consultant/Attending > 10 years

Consultant/ Attending 5-10 years

Consultant/ Attending <5 years

Fellow post completion of specialist training

Registrar/ Senior Trainee

Junior trainee

7. What is the base of your main practice? *

University/Teaching Hospital

Public Healthcare Hospital

Private Hospital

Other

8. How many new breast cancer cases does your Unit treat per year? *

<50

50-100

100-150

150-300

300-500

>500

I don't know

[https://forms.office.com/pages/designpagev2.aspx?lang=en-US&origin=OfficeDotCom&route=Start&subpage=design&id=bCpreHvSf02cWcwEq](https://forms.office.com/pages/designpagev2.aspx?lang=en-US&amp;origin=OfficeDotCom&amp;route=Start&amp;subpage=design&amp;id=bCpreHvSf02cWcwEq)… 3/15

2022-12-18 10:16 PERforator flaps: Doctor needs In Training and Attitudes. The PERDITA Survey

9. Does your Unit have access to free flap plastic surgery service? *

Yes

No

There is plastic surgery service, but no free flaps are performed

10.

11.

What type of procedures do you perform independently? (Multiple answers are allowed) *

I do traditional wide local excisions (lumpectomy) and mastectomies

Wide local excision through remote ("smart") incision placement without tissue rearrangement, but simple glandular approximation(periareolar scar, donut mastopexy, inframammary fold access and so on)

Therapeutic mammaplasty with tissue rearrangement (including, but not limited to mastopexy, breast reduction or different mammaplasty techniques)

Volume replacement with chest wall perforator flaps

Implant based reconstruction for mastectomy

Pedicled flap autologous reconstruction (eg LD, TRAM, etc) for mastectomy

Free flap autologous reconstruction (eg DIEP, TUG, etc) for mastectomy

I do not perform any procedures independently

Have you attended any web-based events or webinars (eg the ibreastbook or other) on chest wall perforator flap reconstruction? *

Yes

No

[https://forms.office.com/pages/designpagev2.aspx?lang=en-US&origin=OfficeDotCom&route=Start&subpage=design&id=bCpreHvSf02cWcwEq](https://forms.office.com/pages/designpagev2.aspx?lang=en-US&amp;origin=OfficeDotCom&amp;route=Start&amp;subpage=design&amp;id=bCpreHvSf02cWcwEq)… 4/15

2022-12-18 10:16 PERforator flaps: Doctor needs In Training and Attitudes. The PERDITA Survey

12.

Have you attended any dedicated courses or workshops (eg., the Cambridge workshop or other) on chest wall perforator flap reconstruction? *

Yes

No

[https://forms.office.com/pages/designpagev2.aspx?lang=en-US&origin=OfficeDotCom&route=Start&subpage=design&id=bCpreHvSf02cWcwEq](https://forms.office.com/pages/designpagev2.aspx?lang=en-US&amp;origin=OfficeDotCom&amp;route=Start&amp;subpage=design&amp;id=bCpreHvSf02cWcwEq)… 5/15

2022-12-18 10:16 PERforator flaps: Doctor needs In Training and Attitudes. The PERDITA Survey

Which sentence describes your service in relation to chest wall perforator flaps:

Please provide answers for the following

13.

Did you intend on learning to do flaps already before attending any educational activity?

I was intending to start with a chest wall perforator flap service in my hospital, and the event motivated me more to start the service.

I was interested in starting with a chest wall perforator flap service and the event dissuaded me from taking this further.

I was not intending to start chest wall perforator flaps, but after attending the event I am now interested

I was not intending to start chest wall perforator flaps and after the event I am still not interested.

I am already doing chest wall perforator flaps and wanted to see what other do and learn new tips and tricks.

I have not attended any online events, but I am interested in starting a new chest wall perforator flap service locally.

I have not attended any online events, and I am not interested in starting a new chest wall perforator flap service locally.

[https://forms.office.com/pages/designpagev2.aspx?lang=en-US&origin=OfficeDotCom&route=Start&subpage=design&id=bCpreHvSf02cWcwEq](https://forms.office.com/pages/designpagev2.aspx?lang=en-US&amp;origin=OfficeDotCom&amp;route=Start&amp;subpage=design&amp;id=bCpreHvSf02cWcwEq)… 6/15

2022-12-18 10:16 PERforator flaps: Doctor needs In Training and Attitudes. The PERDITA Survey

If you are already doing chest wall perforator flaps:

Please provide answers for the following. If you are NOT doing chest wall perforator flaps, then you should not answer questions 13-16.

14.

How many years have you been doing perforator flaps (approximate number)?

15. How many cases per year (approximate number)?

16.

17.

In your experience with chest wall perforator flaps, how do you rate your satisfaction with outcomes?

(tick the number you feel suits best, from "1" for the worst and "10" for the best)

1 2 3 4 5 6 7 8 9 10

In your experience with chest wall perforator flaps, how do you rate the patients' satisfaction with outcomes?

(tick in the number you feel suits best, from "1" for the worst and "10" for the best)

1 2 3 4 5 6 7 8 9 10

[https://forms.office.com/pages/designpagev2.aspx?lang=en-US&origin=OfficeDotCom&route=Start&subpage=design&id=bCpreHvSf02cWcwEq](https://forms.office.com/pages/designpagev2.aspx?lang=en-US&amp;origin=OfficeDotCom&amp;route=Start&amp;subpage=design&amp;id=bCpreHvSf02cWcwEq)… 7/15

2022-12-18 10:16 PERforator flaps: Doctor needs In Training and Attitudes. The PERDITA Survey

Standardization of training in perforator flaps

In the following section, you will be asked to respond to a series of questions regarding your views on how training for flap surgery should be pursued/standardized. If you are already performing flaps, your response may either be how you trained or how you think an optimal training curriculum should look like, if you do not feel that your training is optimal. If you are not performing perforator flaps, your response should describe best your view on the topic.

18. Is it necessary that every breast unit has a perforator flap service? *

Yes

No

There should be referral units

19.

Is it necessary that every breast surgeon should independently perform perforator flap reconstruction? *

Yes

No

There should be specialized surgeons in referral units

20. To learn how to perform perforator flaps, I needed/would need to: *

Travel to a referral centre, overseas/to another country, as this option is not available in my country

Travel to a referral centre in my own country, as the service is not available in my Unit

Train in my own Unit with a trainer (proctor) from another centre, as the service is not available in my Unit

Train in my own Unit, as it provides perforator flap service

[https://forms.office.com/pages/designpagev2.aspx?lang=en-US&origin=OfficeDotCom&route=Start&subpage=design&id=bCpreHvSf02cWcwEq](https://forms.office.com/pages/designpagev2.aspx?lang=en-US&amp;origin=OfficeDotCom&amp;route=Start&amp;subpage=design&amp;id=bCpreHvSf02cWcwEq)… 8/15

2022-12-18 10:16 PERforator flaps: Doctor needs In Training and Attitudes. The PERDITA Survey

How confident do you feel with your current status of knowledge regarding...

(please tick the number you feel fits best with "1" corresponding to "Not at all" and "10" to "Completely"

21. ...chest wall perforator flap anatomy? *

1 2 3 4 5 6 7 8 9 10

22. ...chest wall perforator flap technique? *

1 2 3 4 5 6 7 8 9 10

23.

... indications and contra-indications regarding chest wall perforator flap reconstruction? *

1 2 3 4 5 6 7 8 9 10

24. ... chest wall perforator flap outcomes? *

1 2 3 4 5 6 7 8 9 10

[https://forms.office.com/pages/designpagev2.aspx?lang=en-US&origin=OfficeDotCom&route=Start&subpage=design&id=bCpreHvSf02cWcwEq](https://forms.office.com/pages/designpagev2.aspx?lang=en-US&amp;origin=OfficeDotCom&amp;route=Start&amp;subpage=design&amp;id=bCpreHvSf02cWcwEq)… 9/15

2022-12-18 10:16 PERforator flaps: Doctor needs In Training and Attitudes. The PERDITA Survey

How certain are you that there is literature/resources regarding...

(please tick the number you feel fits best with "1" corresponding to "Not at all" and "10" to "Completely"

25. ...chest wall perforator flap anatomy? *

1 2 3 4 5 6 7 8 9 10

26. ...chest wall perforator flap technique? *

1 2 3 4 5 6 7 8 9 10

27.

... indications and contra-indications regarding chest wall perforator flap reconstruction? *

1 2 3 4 5 6 7 8 9 10

28. ... chest wall perforator flap outcomes? *

1 2 3 4 5 6 7 8 9 10

[https://forms.office.com/pages/designpagev2.aspx?lang=en-US&origin=OfficeDotCom&route=Start&subpage=design&id=bCpreHvSf02cWcwE](https://forms.office.com/pages/designpagev2.aspx?lang=en-US&amp;origin=OfficeDotCom&amp;route=Start&amp;subpage=design&amp;id=bCpreHvSf02cWcwE)… 10/15

2022-12-18 10:16 PERforator flaps: Doctor needs In Training and Attitudes. The PERDITA Survey

Training priorities in chest wall perforator flap reconstruction

[https://forms.office.com/pages/designpagev2.aspx?lang=en-US&origin=OfficeDotCom&route=Start&subpage=design&id=bCpreHvSf02cWcwE](https://forms.office.com/pages/designpagev2.aspx?lang=en-US&amp;origin=OfficeDotCom&amp;route=Start&amp;subpage=design&amp;id=bCpreHvSf02cWcwE)… 11/15

2022-12-18 10:16 PERforator flaps: Doctor needs In Training and Attitudes. The PERDITA Survey

29. Do you need training in... *

Yes No Unsure

...identifying appropriate candidates?

 ...flap markings/des ign?

...basic breast ultrasound?

...basic vascular ultrasound (color doppler)?

...finding / identifying perforators from preoperative radiology?

...raising flaps?

...identifying/ dissecting perforators intraoperative

ly?

...how to
place the flap in the resection cavity?

...closing the wound?

[https://forms.office.com/pages/designpagev2.aspx?lang=en-US&origin=OfficeDotCom&route=Start&subpage=design&id=bCpreHvSf02cWcwE](https://forms.office.com/pages/designpagev2.aspx?lang=en-US&amp;origin=OfficeDotCom&amp;route=Start&amp;subpage=design&amp;id=bCpreHvSf02cWcwE)… 12/15

2022-12-18 10:16 PERforator flaps: Doctor needs In Training and Attitudes. The PERDITA Survey

30.

31.

What would you optimally need before starting doing perforator flaps? (Multiple answers are allowed) *

Read about anatomy/technique/other

Watch videos

Watch more dedicated webinars

Attend a course

Attend a workshop (cadaveric, hands-on, etc)

Attend/assist in theatre

Perform cases supervised

Just start performing cases unsupervised

Please rank in importance for you below, the options from the previous question.

*

Read about anatomy/technique/other

Watch videos

Watch more dedicated webinars

Attend a course

Attend a workshop (cadaveric, hands-on, etc)

Attend/assist in theatre

Perform cases supervised

Just start performing cases unsupervised

[https://forms.office.com/pages/designpagev2.aspx?lang=en-US&origin=OfficeDotCom&route=Start&subpage=design&id=bCpreHvSf02cWcwE](https://forms.office.com/pages/designpagev2.aspx?lang=en-US&amp;origin=OfficeDotCom&amp;route=Start&amp;subpage=design&amp;id=bCpreHvSf02cWcwE)… 13/15

2022-12-18 10:16 PERforator flaps: Doctor needs In Training and Attitudes. The PERDITA Survey

32.

Do you feel you would benefit from a technical, comprehensive "how- to-do-it" academic paper? *

Yes

No

Maybe

It would be unnecessary. There are already papers that have helped me perform the technique

[https://forms.office.com/pages/designpagev2.aspx?lang=en-US&origin=OfficeDotCom&route=Start&subpage=design&id=bCpreHvSf02cWcwE](https://forms.office.com/pages/designpagev2.aspx?lang=en-US&amp;origin=OfficeDotCom&amp;route=Start&amp;subpage=design&amp;id=bCpreHvSf02cWcwE)… 14/15

2022-12-18 10:16 PERforator flaps: Doctor needs In Training and Attitudes. The PERDITA Survey

Mainly for trainees or young consultants/attending/specialist surgeons

Please respond to the following

33.

34.

35.

Does/did your training curriculum include chest wall perforator flap reconstruction?

Yes

No

Unsure

If yes, how many procedures are you required to attend and/or perform (by logbook)? (provide number)

Do you think you will obtain/have obtained the necessary experience during your training?

Yes

No

Unsure

This content is neither created nor endorsed by Microsoft. The data you submit will be sent to the form owner.


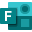
Microsoft Forms

[https://forms.office.com/pages/designpagev2.aspx?lang=en-US&origin=OfficeDotCom&route=Start&subpage=design&id=bCpreHvSf02cWcwE](https://forms.office.com/pages/designpagev2.aspx?lang=en-US&amp;origin=OfficeDotCom&amp;route=Start&amp;subpage=design&amp;id=bCpreHvSf02cWcwE)… 15/15

**Supplementary Figures and Tables**

**Figure S1**


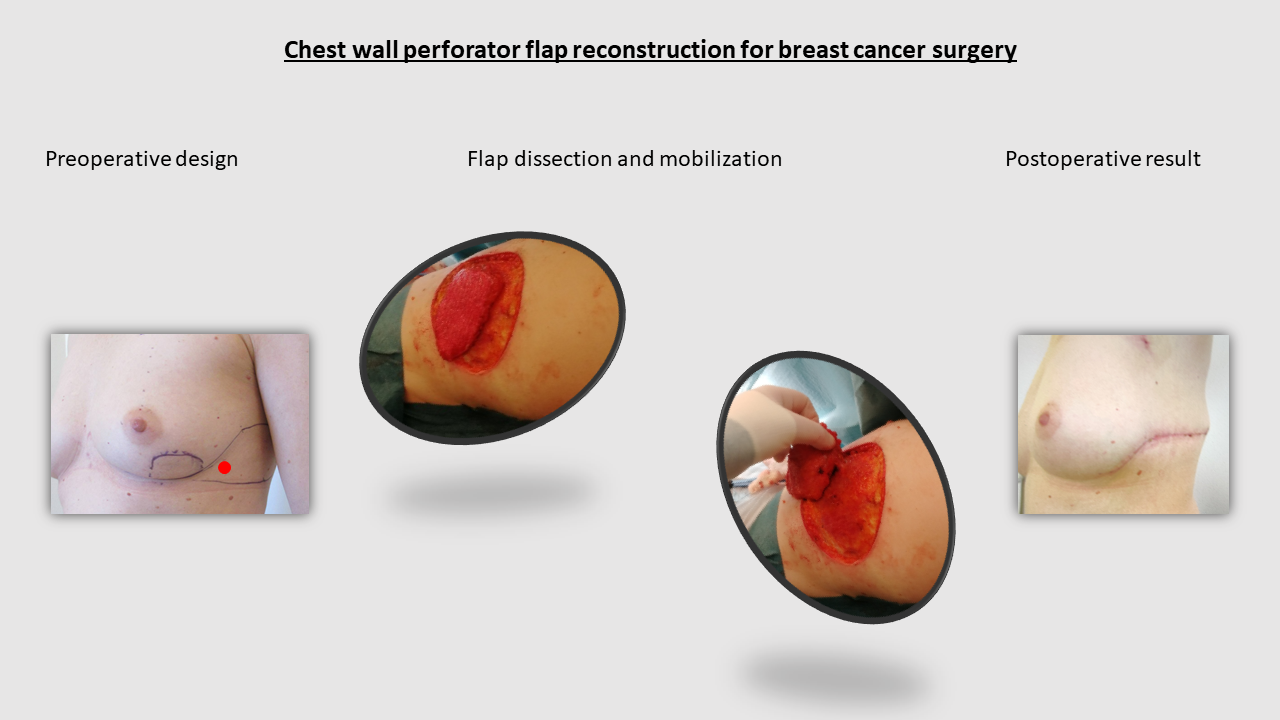


Legend: A patient with a lower-outer quadrant tumour of the left breast (marked area in the breast). The flap has been marked and the red dot demonstrates the perforator site, identified by Doppler ultrasonography. The wide local excision is performed through the lateral breast crease and the flap is de-epithelialized and dissected free. The flap is raised from the chest wall attached only from the vascular pedicle and rotated in the defect.

**Table S1**

Characteristics of survey population.

|  | N | % |
| --- | --- | --- |
| *Region* |  |  |
| Europe | 273 | 44.1 |
| Central Asia | 44 | 7.1 |
| Middle East and North Africa | 84 | 13.6 |
| Sub-Saharan Africa | 14 | 2.3 |
| South Asia | 58 | 9.4 |
| East Asia and Pacific | 46 | 7.4 |
| North America | 13 | 2.1 |
| Latin America and the Caribbean | 67 | 10.8 |
| Not provided/ Missing | 20 | 3.2 |
|  |  |  |
| *Country Income* |  |  |
| High | 304 | 49.1 |
| Upper Middle | 157 | 25.4 |
| Lower Middle | 139 | 22.5 |
| Low | 7 | 1.1 |
| Not provided | 12 | 1.9 |
|  |  |  |
| *Surgical Specialty* |  |  |
| Dedicated Breast Surgeon without any other commitments | 270 | 43.6 |
| General Surgeon doing some breast surgery | 152 | 24.6 |
| Plastic Surgeon | 86 | 13.9 |
| Surgical Oncologist | 85 | 13.7 |
| Gynaecologist | 26 | 4.2 |
|  |  |  |
| *Level of experience* |  |  |
| Consultant/Attending > 10 years | 268 | 43.3 |
| Consultant/ Attending 5-10 years | 123 | 19.9 |
| Consultant/ Attending <5 years | 96 | 15.5 |
| Fellow post-completion of specialist training | 61 | 9.9 |
| Registrar/ Senior Trainee | 47 | 7.6 |
| Junior Trainee | 24 | 3.9 |
|  |  |  |
| *Practice setting* |  |  |
| University/Teaching Hospital | 317 | 51.2 |
| Public Healthcare Hospital | 168 | 27.1 |
| Private Hospital | 126 | 20.4 |
| More than one or all of the above | 7 | 1.1 |
| Missing data | 1 | 0.2 |
|  |  |  |
| *Annual Unit caseload* |  |  |
| <50 | 93 | 15.0 |
| 50-100 | 99 | 16.0 |
| 100-150 | 79 | 12.8 |
| 150-300 | 146 | 23.6 |
| 300-500 | 95 | 15.3 |
| >500 | 87 | 14.1 |
| I don't know | 20 | 3.2 |
|  |  |  |
| *Unit with free flap reconstruction service* |  |  |
| Yes | 315 | 50.9 |
| No | 304 | 49.1 |
|  |  |  |
| *Unit with chest wall perforator service* |  |  |
| Yes | 163 | 26.3 |
| No | 456 | 73.7 |
|  |  |  |
| *Formal oncoplastic training* |  |  |
| Yes | 345 | 55.7 |
| No | 274 | 44.3 |

**Table S2.**

|  | Cluster 1 | Cluster 2 | In-cluster predictor importance |
| --- | --- | --- | --- |
| Cluster Size | 331 | 272 |  |
| Unit with CWPF | Yes (55.5%) | No (97.9%) | 1.00 |
| Performing CWPF independently | Yes (65.1%) | No (92.1%) | 0.99 |
| Surgical Specialty | Dedicated Breast Surgeon (73.5%) | General Surgeon (39.3%) | 0.78 |
| Annual Unit Caseload | 300-500 (28.3%) | <50 (25.7%) | 0.76 |
| Formal Oncoplastic training | No (60.7%) | Yes (77.6%) | 0.42 |
| Region | Europe (60.7%) | Europe (32.6%) | 0.23 |
| Level of experience | Consultant/Attending >10 yrs (50.7%) | Consultant/Attending >10 yrs (38.1%) | 0.10 |
| Practice Setting | University/Teaching Hospital (60.3%) | University/Teaching Hospital (43.2%) | 0.08 |

CWPF: Chest Wall Perforator Flaps

**Table S3.**

| **"Should every Unit offer CWPF?" * "Should every surgeon be able to perform CWPF?" Crosstabulation** | | | | | | |
| --- | --- | --- | --- | --- | --- | --- |
|  | | | "Should every surgeon be able to perform CWPF?" | | | Total |
|  |  |  | No | Yes | There should be specialized surgeons in referral units |  |
| "Should every Unit offer CWPF?" | No | Count | 22 | 13 | 4 | 39 |
|  |  | % within "Should every Unit offer CWPF?" | 56,4% | 33,3% | 10,3% | 100,0% |
|  |  | % within "Should every surgeon be able to perform CWPF?" | 22,9% | 4,0% | 2,0% | **6,3%** |
|  | Yes | Count | 47 | 294 | 74 | 415 |
|  |  | % within "Should every Unit offer CWPF?" | 11,3% | 70,8% | 17,8% | 100,0% |
|  |  | % within "Should every surgeon be able to perform CWPF?" | 49,0% | 90,5% | 37,4% | **67,0%** |
|  | There should be reference centres | Count | 27 | 18 | 120 | 165 |
|  |  | % within "Should every Unit offer CWPF?" | 16,4% | 10,9% | 72,7% | 100,0% |
|  |  | % within "Should every surgeon be able to perform CWPF?" | 28,1% | 5,5% | 60,6% | **26,7%** |
| Total | | Count | 96 | 325 | 198 | 619 |
|  |  | % within "Should every Unit offer CWPF?" | **15,5%** | **52,5%** | **32,0%** | 100,0% |
|  |  | % within "Should every surgeon be able to perform CWPF?" | 100,0% | 100,0% | 100,0% | 100,0% |

Mc Nemar’s Bowker test, p<0.001. CWPF: Chest wall perforator flaps.

**Table S4.**

|  |  | Surgeon performing CWPF (n,%) | | p-value |
| --- | --- | --- | --- | --- |
|  |  | Yes | No |  |
| Anatomy |  |  |  | <0.001 |
|  | “Surgeon better” | 64 (30.6) | 93 (22.7) |  |
|  | “Tie” | 81 (38.8) | 129 (31.5) |  |
|  | “Literature better” | 64 (30.6) | 188 (45.8) |  |
|  | | | | |
| Technique |  |  |  | <0.001 |
|  | “Surgeon better” | 56 (26.8) | 62 (15.1) |  |
|  | “Tie” | 81 (38.7) | 113 (27.6) |  |
|  | “Literature better” | 72 (34.5) | 235 (57.3) |  |
|  | | | | |
| Indications |  |  |  | <0.001 |
|  | “Surgeon better” | 90 (43.1) | 85 (20.7) |  |
|  | “Tie” | 83 (39.7) | 139 (33.9) |  |
|  | “Literature better” | 36 (17.2) | 186 (45.4) |  |
|  | | | | |
| Outcomes |  |  |  | <0.001 |
|  | “Surgeon better” | 87 (41.6) | 86 (21.0) |  |
|  | “Tie” | 81 (38.8) | 158 (38.5) |  |
|  | “Literature better” | 41 (19.6) | 166 (40.5) |  |

The difference of the Likert items (possible range 0-10) ” how would you rate your own knowledge on…” vs “how would you rate the adequacy of literature/learning” was calculated for all four domains explored (anatomy, technique, indications, outcomes). The median differences were categorized as sums in three possible scenarios: “Surgeon better” (respondents scoring their perceived knowledge higher than what they perceive as available literature/sources); “Tie” (respondent scoring equally) and “Literature better” (respondents scoring their perceived knowledge lower than what they perceive as available literature/sources). The numbers correspond to responses and the % to the respective column, as to detect difference in response distribution depending on whether the respondents performed chest wall perforator flaps (CWPF). p-value denotes Chi-square, 2-sided test.

**Table S5**

| ”Do you need training in…” | | Surgeon performing CWPF (n,%) | | p-value* |
| --- | --- | --- | --- | --- |
|  |  | No | Yes |  |
| …identifying CWPF candidates? | No | 64 (15.6) | 101 (48.3) | <0.001 |
|  | Yes | 311 (75.9) | 88 (42.1) |  |
|  | Unsure | 35 (8.5) | 20 (9.6) |  |
| …flap design/markings? | No | 31 (7.6) | 90 (43.1) | <0.001 |
|  | Yes | 370 (90.2) | 107 (51.2) |  |
|  | Unsure | 9 (2.2) | 12 (5.7) |  |
| …basic ultrasound? | No | 121 (29.2) | 101 (48.3) | <0.001 |
|  | Yes | 267 (65.1) | 100 (47.8) |  |
|  | Unsure | 22 (5.4) | 8 (3.8) |  |
| …vascular (Doppler) ultrasound? | No | 74 (18.0) | 77 (36.8) | <0.001 |
|  | Yes | 320 (78.0) | 123 (58.9) |  |
|  | Unsure | 16 (4.0) | 9 (4.3) |  |
| … radiology review for perforator identification? | No | 40 (9.8) | 65 (31.1) | <0.001 |
|  | Yes | 343 (83.7) | 127 (60.8) |  |
|  | Unsure | 27 (6.6) | 17 (8.1) |  |
| … raising a flap? | No | 54 (13.2) | 103 (49.3) | <0.001 |
|  | Yes | 328 (80.0) | 93 (44.5) |  |
|  | Unsure | 28 (6.8) | 13 (6.2) |  |
| …perforator dissection? | No | 30 (7.3) | 80 (38.3) | <0.001 |
|  | Yes | 363 (88.6) | 118 (56.5) |  |
|  | Unsure | 17 (4.1) | 11 (5.3) |  |
| … to place the flap in the cavity? | No | 64 (15.6) | 105 (50.2) | <0.001 |
|  | Yes | 322 (78.5) | 91 (43.5) |  |
|  | Unsure | 24 (5.9) | 13 (6.2) |  |
| …wound closure? | No | 173 (42.2) | 135 (64.6) | <0.001 |
|  | Yes | 210 (51.2) | 68 (32.5) |  |
|  | Unsure | 27 (6.6) | 6 (2.9) |  |

*: Pearson’s Chi square, n: absolute number, per cent in parenthesis (%) denotes the respective subgroup for each question *in the column*, so as to detect differences in response distribution depending on whether the respondent perform chest wall perforator flaps (CWPF).
